# Localized retinal nerve fiber layer defect in patients with COVID-19

**DOI:** 10.5935/0004-2749.20200109

**Published:** 2024-02-11

**Authors:** Kemal Örnek, Emine Temel, Nazife Aşıkgarip, Özkan Kocamış

**Affiliations:** 1 Department of Opthalmology, Kırşehir Ahi Evran University School of Medicine, Kırşehir, Turkey; 2 Department of Opthalmology, Kırşehir Ahi Evran Training and Research Hospital, Kırşehir, Turkey

Dear Editor,

The novel coronavirus disease 2019 (COVID-19) is an extremely contagious disease that has been found to cause severe acute respiratory distress syndrome^(^1^)^. Although ocular findings have mostly been limited to the anterior segment^(^2^-^4^)^, studies have shown that viral ribonucleic acid can be detected in the retina of infected individuals^(^5^)^. Accordingly, Marinho et al. had found lesions at the ganglion cell and inner plexiform layers of patients with COVID-19^(^6^)^. Coronaviruses are capable of producing various ocular manifestations, ranging from conjunctivitis, and anterior uveitis to vision-threatening conditions, such as retinitis and optic neuritis^(^3^)^.

**Figure 1:**
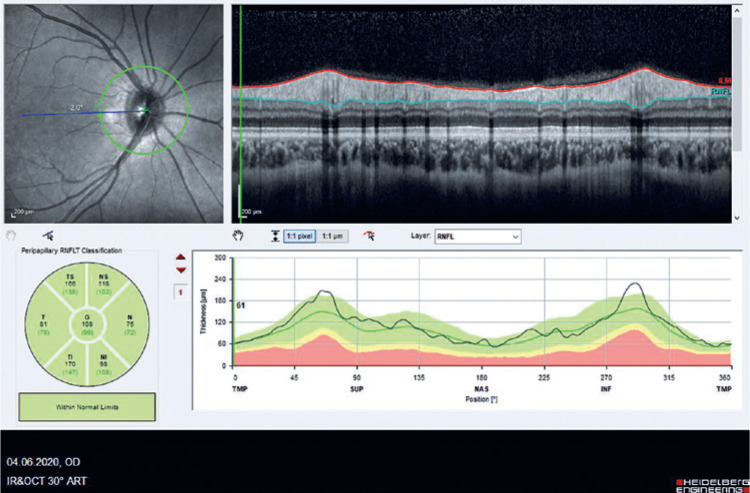
Peripapillary retinal nerve fiber layer measurements using spectral-domain optical coherence tomography.

We evaluated the effect of COVID-19 infection on the peripapillary retinal nerve fiber layer (pRNFL) using spectral-domain optical coherence tomography (SD-OCT) (Figure 1). Our study had been approved by the institutional review board and was performed in accordance with the tenets of the Declaration of Helsinki.

A total of 32 eyes from 32 patients with COVID-19 (Group 1) and 34 eyes from 34 healthy subjects (Group 2) were included. All patients in Group 1 were positive for COVID-19 following real-time reverse transcriptase-polymerase chain reaction from nasopharyngeal swabs. No significant difference in age and gender had been observed between both groups (p=0.6 and 0.4, respectively), while slit-lamp examination was normal for all cases. The average pRNFL thickness values are presented in t able 1. Accordingly, a significant difference in the inferonasal sector had been observed between both groups (p=0.04).

COVID-19 infection is not merely a respiratory system disease; it can be neuroinvasive and cause direct central nervous system infection^(^7^)^. Accordingly, this disease utilizes the angiotensin-converting enzyme 2 (ACE2) receptors to infiltrate in the intracellular space. One report found that the brain expresses ACE2 receptors, which have been detected in glial cells and neurons^(^8^)^, while another documented evidence of viral particles in the neurons and capillary endothelial cells of the frontal lobe^(^9^)^.

The RNFL of the retina contains the non-myelinated axons of retinal ganglion cells that form the optic nerve. Depending on the physiological parameters of RNFL, localized defects are usually more frequent in the temporal inferior fundus region and temporal superior region. Our study found a significant thinning in the inferonasal sector in patients with COVID-19. However, none of patients had coexisting retinopathy or optic nerve changes and a history of optic neuropathy or glaucoma.

Our findings suggest that subclinical damage may occur in patients with COVID-19, which may be localized rather than diffuse axonal loss. To best of our knowledge, this has been the first study to compare pRNFL thickness between patients with COVID-19 and healthy controls. As such, localized RNFL defects that can be assessed by noninvasive SD-OCT imaging may be added to the retinal features of COVID-19.

**Table 1:** Average peripapillary retinal nerve fiber layer thickness values (µm) in all sectors

	Group 1	Group 2
Nasal quadrant	75.55 ± 11.84	77.21 ± 11.31
Inferonasal quadrant	111.97 ± 17.58	121.65 ± 20.47
Temporal quadrant	73.58 ± 9.69	74.21 ± 8.36
Inferotemporal quadrant	146.94 ± 18.89	147.79 ± 15.73
Superotemporal quadrant	141.42 ± 21.49	143.42 ± 24.18
Superonasal quadrant	105.29 ± 18.22	106.38 ± 16.11
